# Patients with ovarian carcinoma excrete different altered levels of urine CD59, kininogen-1 and fragments of inter-alpha-trypsin inhibitor heavy chain H4 and albumin

**DOI:** 10.1186/1477-5956-8-58

**Published:** 2010-11-17

**Authors:** Siti S Abdullah-Soheimi, Boon-Kiong Lim, Onn H Hashim, Adawiyah S Shuib

**Affiliations:** 1 Institute of Biological Sciences, Faculty of Science, University of Malaya, Kuala Lumpur, Malaysia; 2 Department of Obstetrics and Gynaecology, Faculty of Medicine, University of Malaya, Kuala Lumpur, Malaysia; 3 Department of Molecular Medicine, Faculty of Medicine, University of Malaya, Kuala Lumpur, Malaysia; 4 University of Malaya Centre for Proteomics Research, University of Malaya, Kuala Lumpur, Malaysia

## Abstract

**Background:**

Diagnosis of ovarian carcinoma is in urgent need for new complementary biomarkers for early stage detection. Proteins that are aberrantly excreted in the urine of cancer patients are excellent biomarker candidates for development of new noninvasive protocol for early diagnosis and screening purposes. In the present study, urine samples from patients with ovarian carcinoma were analysed by two-dimensional gel electrophoresis and the profiles generated were compared to those similarly obtained from age-matched cancer negative women.

**Results:**

Significant reduced levels of CD59, kininogen-1 and a 39 kDa fragment of inter-alpha-trypsin inhibitor heavy chain H4 (ITIH4), and enhanced excretion of a 19 kDa fragment of albumin, were detected in the urine of patients with ovarian carcinoma compared to the control subjects. The different altered levels of the proteins were confirmed by Western blotting using antisera and a lectin that bind to the respective proteins.

**Conclusion:**

CD59, kininogen-1 and fragments of ITIH4 and albumin may be used as complementary biomarkers in the development of new noninvasive protocols for diagnosis and screening of ovarian carcinoma.

## Background

Ovarian carcinoma is the leading cause of death among gynaecologic malignancy. It is the fourth most common cancer affecting women in Malaysia [1]. Patients with ovarian carcinoma often presented themselves at an advance stage of cancer mainly because of the lack of biomarker for early diagnosis and that the cancer is usually asymptomatic at the early stages [2]. Once the cancer is detected at the advance stage, the five-year survival rate of the patients decreases to 25% even when appropriate treatments were provided [3,4].

The gel-based proteomic analysis provides a convenient method to compare the levels of proteins in bodily fluid samples. In the search for new protein biomarker candidates with clinical diagnostic value, substantial progress has been made in the proteomic analysis of serum samples of patients with different cancers [5-7]. In contrast, fewer studies have been carried out on the urine samples of cancer patients. This is despite that urine is generally a better sample for investigative and screening purposes and that the use of urine protein biomarkers such as albumin and human chorionic gonadotropin for clinical diagnosis has been a long standing practice.

The proteomic analysis of urine offers ample opportunities for clinical translation [8,9]. To date, proteomic experiments that have been conducted on urine were not confined to patients suffering from diseases of the genitourinary system [10] but were also carried out on those with atherosclerosis [11], sleep disorder [12] and cancers of the bladder [13], pancreas [14,15], lung [16] and colon [17]. Proteomic investigation has been performed on urine of patients with ovarian carcinoma but is currently restricted to the low molecular weight peptide analysis using the SELDI-TOF-MS approach [18].

In the present study, urine protein samples from patients with ovarian carcinoma and cancer negative women were subjected to the conventional two-dimensional electrophoresis (2-DE) and densitometry analysis. Proteins that were aberrantly excreted by the cancer patients, relative to control subjects, were identified by mass spectrometry and their altered levels in the patients urine were confirmed by Western blotting using antisera and a lectin that bind to the respective proteins.

## Results

### 2-DE profiles of urine proteins

**Figure 1:**
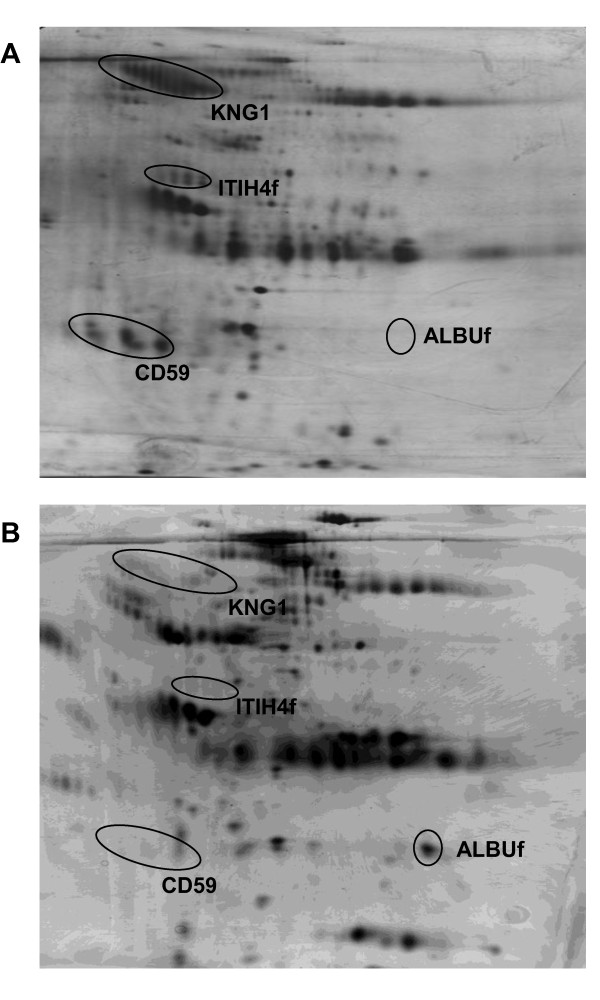
**Typical 2-DE urine protein profiles of controls and patients with ovarian cancer**. Panels A and B demonstrate the representative 2-DE urine protein profiles of control subjects and ovarian cancer patients, respectively. The aberrantly excreted urine protein spot clusters were marked in circles. KNG1, ITIH4f and ALBUf refer to kininogen-1 and fragments of inter-alpha-trypsin inhibitor heavy chain H4 and albumin, respectively. Acid side of 2-DE gel is to the left and relative molecular mass declines from the top.

Separation of urine protein samples by 2-DE resulted in highly resolved profiles comprising more than ten clusters of protein spots. Panel A of Figure 1 demonstrates a representative urinary proteome profile obtained from a control subject. Seven protein spot clusters consistently appeared in all the 15 control samples analyzed and there was no apparent difference in the intensity of the spots between the individual urine samples studied. When the gel-based proteomic analysis was performed on urine protein samples from patients with ovarian carcinoma (n = 11), different 2-DE profiles were obtained (Figure 1, panel B). Three protein spot clusters which consistently appeared in the control profile were either not detected or were reduced in intensity in the cancer patients while one protein spot appeared enhanced in a considerable number of the patients' 2-DE gels. The levels of the other protein spot clusters were comparable to those detected in the urinary proteome profiles of the control subjects.

### Identification of aberrantly excreted urine proteins

Subjecting the spot clusters of urine proteins that were aberrantly excreted to mass spectrometry and database search identified them as CD59, kininogen-1, inter-alpha-trypsin inhibitor heavy chain H4 (ITIH4) and albumin. Table 1 shows a summary of the data acquired. High probability-based MOWSE scores were obtained for all the urine proteins. Among the four urine proteins of interest, ITIH4 and albumin demonstrated large discrepancies between the experimental masses that were estimated based on their mobilities in the 2-DE gels and their theoretically calculated mass. This suggested that the ITIH4 and albumin spots detected in the 2-DE urinary profiles were truncated fragments of their native molecules.

**Table 1:** Mass spectrometric identification of spot clusters from urine protein profiles.

Protein	Accession **number**^#^	Nominal mass (kDa)/p*I*	Mean Experimental mass* (kDa)	MOWSE protein score	Sequence coverage (%)
CD59	P13987	14.168/6.02	19.43	160	18
KNG1	P01042	71.912/6.34	64.34	193	15
ITIH4	Q14624	103.262/6.51	38.85	120	7
ALBU	P02768	69.321/5.92	19.45	161	8

Spot ID are as in Fig. 1. KNG1, ITIH4 and ALBU refer to kininogen-1, inter-alpha-trypsin inhibitor heavy chain H4 and albumin, respectively.

^# ^Accession numbers are from the Mascot database http://www.matrixscience.com.

*Estimation based on migration in the 2-DE gels relative to protein markers.

In the case of ITIH4 (Q14624), the peptide sequences identified with high confidence from the MS/MS correlated to the C-terminal region of the protein, when they were checked against the Swiss-Prot database (Table 2). Sequences obtained were those that spanned within the kallikrein-generated 35 kDa fragment region of ITIH4 (amino acids 696-930). However, molecular mass estimation based on its relative mobility in 2-DE gels indicated a larger fragment of approximately 39 kDa. In case of albumin (P02768), the sequences derived from the MS/MS analysis were confined to amino acids 118 to 281 of the molecule (Table 2).

**Table 2:** List of matched peptide sequences of high confidence identified from MS/MS analysis.

Peptide sequence	Ion score	Amino acid
*39 kDa ITIH4 spots*		
R.QGPVNLLSDPEQGVEVTGQYER.E	19	754 - 775
K.WKETLFSVMPGLK.M	31	814 - 826
R.RLDYQEGPPGVEISCWSVEL.-	24	911 - 930
		
*19 kDa albumin spots*		
K.QEPERNECFLQHK.D	30	118 - 130
K.DDNPNLPR.L	37	131 - 138
K.YLYEIAR.R	43	162 - 168
K.LDELRDEGK.A	10	206 - 214
K.VHTECCHGDLLECADDR.A	41	265 - 281

Sequences of peptide were checked against ITIH4 (Q14624; 930 amino acids) and albumin (P02768; 585 amino acids) in the Swiss-Prot database.

### Image analysis of 2-DE gels

**Figure 2:**
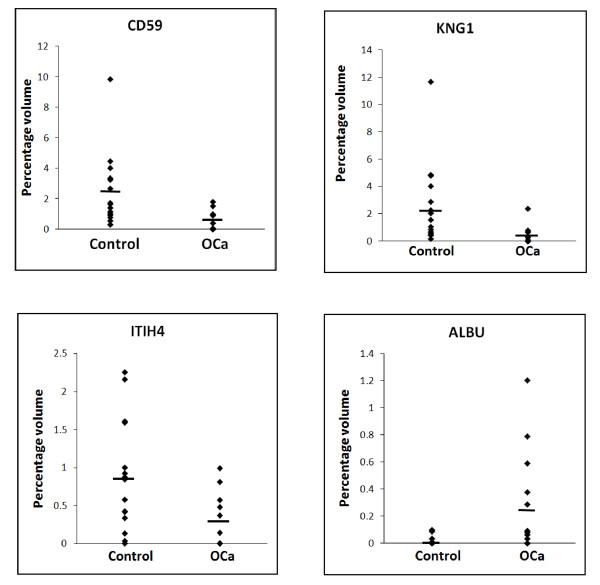
**Relative excretion of urine proteins by control subjects and patients with ovarian cancer**. The percentage of volume contribution was determined using the Image Master 2D Platinum Software 7.0. Image analysis performed on protein spot clusters that appeared consistently within each cohort of urine samples demonstrated the aberrant excretion of CD59, kininogen-1 (KNG1), ITIH4 (39 kDa fragment) and 19 kDa fragment of albumin (ALBUf) by patients with ovarian carcinoma (OCa).

The different altered levels of CD59, kininogen-1 and fragments of ITIH4 and albumin in the urine of patients with ovarian carcinoma, relative to the controls, was confirmed when their 2-DE urine protein profiles were subjected to image analysis using the Image Master 2 D Platinum Software 7.0. Image analysis also confirmed that the levels of the other highly resolved urine protein spot clusters were comparable between cancer patients and controls. Figure 2 demonstrates the mean percentage of volume contribution of the four urine proteins of interest in control subjects and patients with ovarian carcinoma. When taken as overall, the levels of CD59, kininogen-1 and ITIH4 fragment were significantly lower in ovarian carcinoma patients by 3.6-, 2.5- and 1.9-folds, respectively, compared to those excreted by the control subjects. In contrast, the 19 kDa fragment of albumin appeared 274-fold higher in the patients urine (Table 3).

**Table 3:** Relative excretion of urine proteins in ovarian cancer.

Urine proteins	Fold changes*	*p*
CD59	- 3.60	0.001
Kininogen-1	- 2.50	0.001
ITIH4^tf^	- 1.86	0.002
Albumin^tf^	+ 274.07	0.018

*Fold expression changes are relative to the control values

(-) decrease in expression; (+) increase in expression

^tf ^Truncated fragment of protein

A *p *value of less than 0.05 is considered significant.

### SDS-polyacrylamide gel electrophoresis and Western blotting

**Figure 3:**
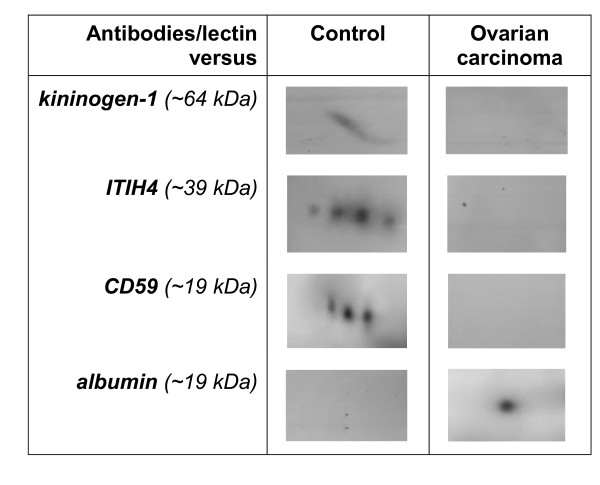
**Interaction of antisera and CGB lectin with aberrantly excreted urine proteins**. Pooled urine samples of ovarian cancer patients (OCa) and those of control subjects (Con) were subjected to SDS-PAGE and Western blotting before being independently exposed to antisera that bind to CD59, kininogen-1 and albumin as well as the ITIH4 binding CGB lectin.

Further confirmation of the altered levels of CD59, kininogen-1 and fragments of ITIH4 and albumin in the urine of patients with ovarian carcinoma relative to those of the control subjects was performed using antibodies and a lectin that bind to the respective proteins that were blotted onto membranes. Figure 3 demonstrates the respective interactions of specific antibodies and the CGB lectin with the four proteins of interest in pooled urine samples of patients and control subjects. In case of the 19 kDa albumin fragment, interaction appeared to be detected only in the pooled urine of patients with ovarian carcinoma compared to that of the controls, while the inverse was observed for CD59, kininogen-1 and the 39 kDa fragment of ITIH4.

## Discussion

In the present proteomic profiling study, the significant reduced excretion of CD59, kininogen-1 and a 39 kDa fragment of ITIH4, and the enhanced levels of a 19 kDa fragment of albumin were detected in the urine samples of patients with ovarian carcinoma relative to those of the control subjects. Their different altered levels in the urine of ovarian cancer patients were confirmed by Western blotting using antisera and a lectin that bind to the respective proteins. These urinary proteins have potential to be used as complementary molecular indicators for noninvasive diagnoses and/or monitoring of ovarian carcinoma, although this requires further confirmation involving a larger scale clinical investigation.

CD59, a cell surface molecule, functions to inhibit the membrane attack complex of the complement pathway. The soluble form of CD59 is usually found in normal human urine at a concentration of about 3.7 μg/ml. However, it is barely detectable in the blood (between 33-119 ng/ml), and even that only in the presence of detergents [19,20]. To the best of our knowledge, the present study is the first to report the decreased levels of CD59 in the urine of patients with ovarian cancer although similar reduced excretion of the protein had previously been reported in the urine of patients with bladder cancer [13] and pancreatic ductal adenocarcinoma [14]. The reason for the low levels of CD59 in the urine of cancer patients is not understood. One possibility is that since the turnover of cancer cells bearing CD59 is low as they are generally "immortal", less of the cell surface molecules are being solubilized and excreted in the urine. However, this remains to be further proven.

Like CD59, kininogen-1 is also detectable in the urine of healthy individuals. Previous studies performed on serum and plasma samples have shown that the expression of kininogen-1 was significantly reduced in patients with gastrointestinal cancer [21], breast cancer [22] and two different types of cervical cancer [23]. Since kininogen-1 is known for its antiangiogenic properties and inhibitory action on the proliferation of endothelial cells [24], its lowered expression in serum/plasma of the cancer patients was believed to have contributed to the survival of the cancer cells [23]. In view of these previous reports, it was not surprising to find similar reduced levels of kininogen-1 in the urine of patients with ovarian carcinoma in this study. However, the aberrant kininogen-1 expression is apparently not cancer-specific since decreased levels of the protein had previously been reported in the urine of patients with chronic pancreatitis [14], interstitial cystitis [25] and IgA nephropathy [26], although the cause for the altered levels of kininogen-1 in these diseases may have been different.

The precise reason for the reduced levels of the ITIH4 fragment in the urine of patients with ovarian carcinoma that is observed in this study is currently not understood. The estimated molecular mass of the urine ITIH4 fragment indicated that it was slightly larger than its reported 35 kDa serum counterpart in the ovarian carcinoma patients [27]. Detection of the different sizes of ITIH4 fragments was not surprising as previous studies using SELDI-TOF-MS have demonstrated that ITIH4 was extensively processed within its proline-rich region in the human serum. In different diseases including ovarian carcinoma, different fragments were shown to be proteolytically generated [28]. While the present study demonstrated the reduced levels of the 39 kDa ITIH4 fragment in the urine of patients with ovarian carcinoma, our previous data showed the up-regulated levels of a 35 kDa ITIH4 fragment in the serum samples of the patients [27]. This inverse relationship and the difference in the molecular masses of the ITIH4 fragments detected in the respective samples suggest presence of a selective glomerular filtration mechanism that retained the 35 kDa fragment in the blood but allowed its 39 kDa counterpart to be excreted in the urine.

Based on their resolved locations in the 2-DE gels and MS/MS derived sequences, the enhanced albumin spots detected in the urine of ovarian cancer patients in this study appeared to be fragments of albumin that consist of amino acids between positions 118 to 281, and with an approximate molecular mass of 19 kDa. The human urine is known to contain low levels of albumin fragments, with some polypeptides containing discontinuous sequences joined by unknown crosslinks [29]. Since the 19 kDa albumin fragment was present only in trace quantities in the urine of the control subjects, it may be used as a complementary urine biomarker to differentiate ovarian carcinoma patients from healthy individuals.

## Conclusion

The proteomic profiling of urine samples demonstrated reduced levels of CD59, kininogen-1 and a 39 kDa ITIH4 fragment, as well as the enhanced excretion of a 19 kDa fragment of albumin in patients with ovarian carcinoma compared to control women. This observation may be applied in the development of noninvasive protocols for diagnosis and/or monitoring of the cancer.

## Methods

### Urine samples and processing

Urine samples were collected from patients newly confirmed with stages II and III ovarian carcinoma (n = 11), prior to treatment, at the University of Malaya Medical Centre (UMMC), Kuala Lumpur. All patients showed normal serum creatinine values. Control urine samples were collected randomly from age-matched cancer negative women (n = 15). Samples obtained were with consent and approval granted by the ethical committee of UMMC in accordance to the ICH GCP guideline and the Declaration of Helsinki. The subjects were of different ethnic background (Malay, Chinese and Indian). Sodium azide was immediately added to the urine upon collection to a final concentration of 20 mM. The samples were centrifuged at 10,000 rpm at 4°C and the supernatant was collected and dialyzed against distilled water. The urine proteins were aliquoted, freeze-dried and kept at -20°C. Protein content was determined using the Pierce BCA protein assay kit (Thermo Fisher Scientific, Rockford USA).

### Two-dimensional gel electrophoresis

IPG strips (pH 3-10, 11 cm) were rehydrated overnight in presence of 300 μg urine proteins in 200 μl rehydration solution (8 M urea, 0.5% v/v Pharmalyte 3-10, 0.5% v/v NP-40). Isoelectric focusing was performed using the Multiphor™ II Electrophoresis unit (GE Healthcare, Uppsala, Sweden) for a total of 12001 Vh at 20°C. The samples were then reduced by incubation of the strips in equilibrium buffer (50 mM Tris-HCl pH 8.8, 6 M urea, 30% glycerol, 2% SDS) containing 1% w/v DTT for 15 min prior to SDS-PAGE, and alkylated using 2.5% w/v iodoacetamide in the same equilibrium buffer for another 15 min. The strips were then laid onto 12.5% polyacrylamide gels and electrophoresis was performed at 25 mA per gel.

### Silver staining and image analysis

The 2-DE gels were developed by silver staining according to the method of Heukeshoven and Dernick [30] and scanned using the Image Scanner III. For mass spectrometric analysis, staining of gels was performed in absence of glutaraldehyde. Protein profiles were evaluated using the ImageMaster™ 2 D Platinum Software (Version 7). Image analysis was restricted to protein spot clusters that appeared consistently within each cohort of urine samples. The levels of proteins in each urine sample were evaluated as a percentage of volume contribution (%vol) to eliminate possible variations due to differential staining.

### Mass spectrometry and database search

Protein spots of interest were excised from the silver stained gels and subjected to in-gel digestion according to the method of Shevchenko *et al. *[31]. Gel plugs were destained using 50 mM sodium thiosulphate: 15 mM potassium ferricyanide (1:1; v/v). Proteins in the plugs were reduced with 10 mM DTT in 100 mM ammonium bicarbonate for 30 min at 60°C, followed by alkylation with 55 mM iodoacetamide in the same solution for 20 min at RT in dark. The gel plugs were washed with 50% acetonitrile (ACN) in 100 mM ammonium bicarbonate, dehydrated by incubating in 50 μl ACN for 15 min and left to dry using a speed vac. Proteins were then digested with 7 ng/μl trypsin in 50 mM ammonium bicarbonate overnight at 37°C, extracted twice using 50% ACN and concentrated using the speed vac. The resulting peptide solutions were desalted and concentrated using zip-tips (Perfect Pure C18, Eppendorf, Hamburg, Germany). One μl aliquot was spotted onto a sample plate with 1 μl of matrix solution (α-cyano-4hydroxycinnamic acid, 10 mg/ml in 70% v/v ACN, 0.1% v/v TFA) and was allowed to air dry.

MALDI mass spectrometry was performed using the Applied Biosystems 4800 Proteomics Analyser. Spectra were initially acquired in reflecton mode in the mass range of 1000 to 4000 Da. The instrument was then switched to MS/MS (TOF/TOF). Ten strongest peptides from the MS scan were isolated, fragmented and reaccelerated to measure their masses and intensities. The data were exported in a format suitable for submission to the MASCOT database search program (Matrix Science Ltd., London, UK) and searched against 'all entries'. Identification was accepted when ≥ 5 peptide masses matched to a particular protein (mass error ± 50 ppm - 1 missed cleavage) and the MOWSE score was over the threshold score at *p *= 0.05.

### SDS-polyacrylamide gel electrophoresis and Western blotting

Urine samples of patients with ovarian carcinoma (n = 11) and control subjects (n = 15) were separately pooled and subjected to unidimensional SDS-PAGE according to the method of Laemmli [32]. Gels consisting of 12.5% w/v acylamide were used. Separated proteins were transferred to nitrocellulose membranes (0.45 μm) using the NovaBlot Kit of Multiphor II Electrophoresis System (GE Healthcare, Uppsala, Sweden) at 0.8 mA/cm^2^.

The membranes were blocked with 3% w/v gelatine in Tris-buffered saline (TBS), pH 7.5, for 1 h at RT and washed three times with the same buffer. They were then incubated for another 1 h in the following HRP-conjugate solutions: (1) anti-human CD59 (Abcam, Cambridge, UK - Cat. No. ab9182, at 1:5 dilution), (2) anti-human kininogen-1 (Abnova, Jhongli, Taiwan - Cat. No. H00003827-B01, at 1:500 dilution), (3) anti-albumin (Sigma Chemical Company, St. Louis, MO USA - Cat. No. A0433, at 1:40 dilution) and (4) champedak galactose binding (CGB) lectin (0.01 μg/ml) diluted/dissolved in TBST. The use of the CGB lectin to detect the C-terminal O-glycosylated ITIH4 fragment has been previously reported [27]. Development of the Western blot was performed using 25 ng 3,3'-diaminobenzidine and 5 μl 30% v/v H_2_O_2 _in 50 ml TBS. The reaction was stopped by washing the membranes with distilled water.

### Statistical analysis

All values are presented as mean ± SD. The Student's *t*-test was used to analyze significance of differences between control subjects and patients. A *p *value of less than 0.05 was considered significant.

## Competing interests

The authors declare that they have no competing interests.

## Authors' contributions

SSAS carried out the experiments, analyzed the data and drafted the manuscript; BKL provided the urine samples; OHH contributed to the design of the study and critically revised the manuscript; ASS planned the study and critically revised the manuscript. All authors read and approved the final manuscript.
